# An AI without ethical boundaries?

**DOI:** 10.17179/excli2024-7375

**Published:** 2024-08-19

**Authors:** Lucindo José Quintans-Júnior

**Affiliations:** 1 Universidade Federal de Sergipe (UFS), Avenida Marcelo Deda Chagas, Bairro, Rosa Elze, CEP 49107-230, São Cristóvão, Sergipe, Brazil

## ⁯⁯

Artificial Intelligence (AI) and machine learning have revolutionized society, accelerating processes and services in unprecedented ways, creating new paradigms and suggesting it could be a new Panacea (Quintans-Júnior et al., 2023[3]). However, the limits that humans impose on AI should ensure that its actions are not harmful to mankind, but a recent revelation about the use of AI by Israel to designate targets in Gaza, resulting in civilian casualties, highlights the danger of AI-driven warfare. The investigation exposed a system called "Lavander", which identified possible Hamas members, leading to airstrikes that caused deaths, including innocent civilians and children (McKernan and Davies, 2024[1]).

Thus, the barrier of "ethical" limits theoretically imposed by the creators of AI is subjugated by individual and collective interests, regardless of whether ethical principles are neglected or intentionally abolished. So, is the limit limitless?

An article published by Yip et al. (2023[4]) described how AI applications in medical robots are ushering in a new era for medicine. In the realm of rehabilitation devices and advanced prosthetics, AI holds promise for delivering personalized assistance, enhancing functionality, and facilitating mobility. The convergence of remarkable progress in robotics, medicine, materials science, and computing stands to revolutionize patient care, potentially offering safer, more effective, and readily accessible treatment options in the times ahead (Yip et al., 2023[4]). However, the case of AI use in Israel makes us reflect on the parameters that will be adopted in AI for medical robots to mitigate the possibility of major ethical conflicts and, more seriously, the exposure of patients' lives, including patients unable to determine the course of their own lives, such as children, some elderly people, and those in an unconscious state.

Standing on the threshold of the future, AI is set to revolutionize medicine, offering both promise and ethical challenges. This potential for unprecedented advancements must be balanced with caution (Quintans-Júnior et al., 2024[2]). As we integrate AI into healthcare, it is vital to ensure that technology enhances rather than replaces human connection. Without moral and ethical constraints, AI's use in medical robots could lead to harmful consequences. Balancing innovation with empathy will shape a humane and effective future for healthcare. 

## Conflict of interest

The author declares no conflicting interests. 
